# Replicating and extending the reliability, criterion validity, and treatment sensitivity of the shortened PANSS for pediatric trials

**DOI:** 10.1007/s00787-025-02681-1

**Published:** 2025-03-10

**Authors:** Joan Busner, Eric A. Youngstrom, Joshua A. Langfus, David G. Daniel, Robert L. Findling

**Affiliations:** 1 Signant Health, Blue Bell, PA USA; 2 Virginia Commonwealth University School of Medicine, Richmond, VA USA; 3 Nationwide Children’s Hospital and The Ohio State University, Helping Give Away Psychological Science, University of North Carolina at Chapel Hill, Chapel Hill, NC USA; 4 University of North Carolina at Chapel Hill, Chapel Hill, NC USA; 5 Signant Health, McLean, VA USA; 6 George Washington University School of Medicine, Washington, DC USA

**Keywords:** Pediatric, Assessment, Schizophrenia, Psychopharmacology, PANSS, Child psychiatry

## Abstract

Do the shortened Positive and Negative Syndrome Scale (PANSS) (Kay et al., J Clin Psychiatry 58:538–546, 1987) versions recently developed from a National Institute of Mental Health (NIMH) pediatric dataset continue to perform well in a third independent randomized double-blind clinical trial of adolescents with schizophrenia? Secondary analysis of the double-blind, placebo-controlled aripiprazole pivotal trial data (*N* = 302) found that the 10-item (and 20-item) PANSS versions on which we have previously reported (Findling et al., J Am Acad Child Adolesc Psychiatry, 10.1016/j.jaac.2022.07.864, 2023) continued to provide high reliability, strong convergent correlation with expected measures, and treatment effects that equaled those found in the 30-item adult PANSS. Our shortened PANSS, derived originally from the randomized non-placebo controlled NIMH Treatment of Early Onset Schizophrenia Spectrum study (TEOSS) (Sikich et al., Am J Psychiatry 165(11):1420–1431, 2008), and independently replicated in both the placebo-controlled paliperidone pivotal trial for adolescents with schizophrenia (Youngstrom et al., PsyArxiv, 10.31234/osf.io/zb695, 2023), and now the placebo-controlled aripiprazole pivotal trial for adolescents with schizophrenia, has again performed as well as the full 30 item adult-patient derived PANSS. The findings suggest it is possible to reduce the PANSS interview by 2 thirds, thus reducing burden on families and pediatric patients as well as administration and training costs, while maintaining high reliability, validity, and sensitivity to treatment equal to that of the 30-item version.

**Supplementary Information:**

The online version contains supplementary material available at 10.1007/s00787-025-02681-1.

## Introduction

In an effort at optimizing the 30-item PANSS [1] for use in pediatric trials, our group empirically derived a psychometrically optimized 10-item version, as well as a 20-item version, as described at length in Findling et al. [2]. The work was met with excitement in the field as highlighted by an accompanying editorial by Benedetto Vitiello in the influential *Journal of the American Academy of Child and Adolescent Psychiatry* entitled, “Can Less Be More When Measuring Psychotic Symptoms in Youth?” [3].

The 10-item version, as well as a 20-item version, was generated using treatment data from the NIMH-funded TEOSS study – a randomized comparison of three (active) antipsychotic agents in youth with schizophrenia [4]. As the TEOSS study did not contain a placebo arm it was important to not only replicate the shortened PANSS findings in an independent sample but to do so in a pediatric sample that ideally contained a placebo arm. Further, as noted in the Vitiello editorial, examining the performance of the shortened PANSS in a pediatric sample that had shown a statistically significant effect of drug vs placebo would allow for detection of the relative treatment sensitivity of the shortened vs the full 30 item versions.

Toward these goals we have subsequently examined our 10-item and 20-item PANSS versions in a large, independent, positive, placebo controlled pivotal registration trial of paliperidone [5] and showed excellent reliability and validity as well as sensitivity to treatment using the 10-item and 20-item versions in comparison to the 30-item versions.

The present study provides the opportunity to again examine the shortened PANSS versions via secondary analyses of a second, independent, placebo-controlled positive phase 3 registration trial in adolescents with schizophrenia: the Otsuka aripiprazole pivotal trial [6].

Specific aims for the present study include evaluating the performance of our 10-item and 20-item versions compared to the full-length 30-item version in terms of statistical model fit, reliability of the subscale scores, content coverage, and calibration against the full-length version, and comparison of convergent validity with secondary outcome measures of functioning and treatment response. As done in the paliperidone dataset, we also evaluated the sensitivity of the scales to time, drug vs placebo treatment, and time by treatment effects, and compared the effect sizes to those generated by the full-length version. As per our previous analyses, we hypothesized that once again a five-factor model would fit best, that the correlation between the short and full-length versions would be high, and that any score bias would be small. In addition, we report precision and change benchmarks that should help facilitate application to individual clinical cases.

## Method

Participant level data from the “Phase 3 Aripiprazole in Adolescents with Schizophrenia (APEX 239) Study” (Clinical Trial Registry number: NCT00102063) were accessed through Vivli, Inc., with permission of Otsuka. The clinical trial study details were reported in Findling et al. [6].

### Measures

#### PANSS

The PANSS is a 30-item interview rating positive (P), negative (N), and general psychopathology (G) cognitive, and affective symptoms often associated with schizophrenia and psychosis using a 1 (*absent*) to 7 (*extreme*) scale [1]. It was developed for adults. In this trial, as usual for pediatric registration trials, trained raters scored all items by separately interviewing both the pediatric patient and the primary caregiver, focusing on the last week. Conventionally all 30 items are summed for a total score. There also are 5 subscales based on factor analyses in multiple samples [2, 5, 7–9]. For the analysis, our scale item assignments for the 10-item and 20-item versions followed the analyses of Findling et al. [2]. Present analyses used item averages rather than sums; these produce identical statistical significance tests, while also scaling consistently (1–7) allowing for comparison of agreement and calibration across scores based on different numbers of items [10]. We refer to the total scores from each scale as the PANSS10, PANSS20, PANSS30 in the results and tables.

#### Criterion validity measures

Study raters also completed the Clinical Global Impressions of Severity (CGI-S) [11] at each visit.

### Statistical analyses

Confirmatory factor analysis (CFA) with ML estimation evaluated fit of the 10-item and 20-item five factor/subscale models developed in Findling et al. [2], as well as the 30-item five factor model [6]. Average item correlation quantified internal consistency independent of number of scale item, and λ [5] and MacDonald’s omega as reliability estimates appropriate for multi-factor composite scales [10], and then item response theory (IRT) to estimate marginal reliability across a range of severity levels, as well as option characteristics for each item [12], analyzing each factor separately. Regression analyses and Bland–Altman plots examined content coverage and calibration [13]. Correlations for the 30-, 20-, and 10-item scores and the CGI-S, both at baseline and across all visits, were tested for differences. GLM tested treatment effects, including partial eta-squared coefficients for time, treatment arm, and time*treatment interaction. Analyses used the *R* packages *psych* (scoring, classical test theory reliability estimates), *lavaan* (CFAs), and *mirt* (IRT).

### Procedure

JB and EAY prepared a secondary analysis request, which the other authors reviewed before submission to Vivli and the University of North Carolina Institutional Review Board. After approval and completion of requisite agreements, the analysis team (JAL, EAY) received secure logins and accessed the data for analysis. Details about the conduct of the clinical trial itself are reported in Findling et al. [6]. Briefly, this was a multicenter placebo-controlled randomized study of 302 13–17 year olds with DSM-IV diagnosed schizophrenia and a required PANSS total score of 70 or greater at baseline. Adolescents were randomized 1:1:1 to one of three arms: 10 mg/day aripiprazole, 30 mg /day aripiprazole, or placebo. The primary outcome was change in PANSS (30-item) total score from baseline to Day 42 or last postbaseline assessment if discontinuing earlier. The study was positive, with significant separation from placebo for each of the two active dose arms. Please see Findling et al. [6] for study details.

## Results

### Participants

A total of 302 adolescents between the ages of 13 and 17 years (mean age = 15.47, *SD* = 1.47 years; 57% male), from 101 treatment centers in the United States, South America, Europe, Asia, South Africa, and the Caribbean were randomized into the 6 week double-blind trial. Baseline data analyses used *N* = 302, and supplemental psychometric analyses of later data either used all available visits or last observation carried forward (LOCF).

### Confirmatory factor analyses

Table 1 reports fit indices for both one- and five-factor models with 10, 20, or 30 items. The five factors (Withdrawal/Apathy, Thought Disturbance, Aggression, Internalizing, and Delusions/Odd Content) were specified a priori based on prior results [1, 5, 6] with the specified loadings indicated in supplemental Tables 1a–c, available online. Analyses based on different item sets are not statistically “nested,” precluding direct comparisons of model fit. CFA model parameterization was identical to that in Findling et al. [2] and Youngstrom et al. [5].

**Table 1:** Comparison of fit indices for confirmatory factor analyses of one and five factor models based on 30, 20, and 10 items (*N* = 302)

Fit Index	30 Items (Marder)	20 Items	10 Items
One factor model			
*X*^*2*^ (*df*)	2236.28 (405)	1293.087 (170)	478.84 (35)
CFI	.442	.472	.348
TLI	.400	.410	.161
RMSEA	.122	.148	.205
SRMR	.132	.147	.137
BIC	28,268.12	18,750.09	9597.29
Five factor model			
*X*^*2*^ (*df*)	1254.61 (367)	572.81 (160)	88.79 (27)
CFI	.715	.806	.909
TLI	.685	.769	.849
RMSEA	.089	.092	.087
SRMR	.103	.086	.069
BIC	26,561.40	18,086.915	9252.93

*df* degrees of freedom, *CFI* Comparative Fit Index, *TLI* Tucker-Lewis Index (higher is better fit), *RMSEA* Root Mean Squared Error of Approximation, *SRMR* Standardized Root Mean square Residual (smaller is better fit), *BIC* Bayesian Information Criterion (lower number indicates the preferable model when comparing two or more)

In all three item sets (Table 1), the performance of the five-factor models surpassed that of the single-factor models. In the 30-item set, the five-factor model exhibited inadequate fit according to all indices, and three items demonstrated only modest loadings (< 0.33) on their respective factors (G12 *Lack of judgment and insight* loaded at 0.17, *G1 Somatic concern* at 0.25, and N7 *Stereotyped thinking* at 0.325). Conversely, the 10 and 20 item five-factor models displayed satisfactory fit (supplemental Table 1), with all items showing significant loadings on the appropriate factors.

### Reliability and precision

Table 2 shows the reliability and precision estimates for the composite scores. The average inter-item correlation was 0.15 for the 10 items and the 30 items, and 0.16 for the 20 items. Omega^Total^ ranged from 0.84 (PANSS10) to 0.90 (PANSS30). Omega^Total^ is conceptually the most appropriate reliability estimate for a total score on the PANSS, as the total is creating a composite sum across five different and only modestly correlated factors [10].

**Table 2:** Reliability, correlation with full-length scale, and length reduction for composite scores (scaled as item averages, ranging from 1 to 7) using baseline data from acute phase (*N* = 302)

*Version*	*PANSS10*	*20-item*	*Full-length*
Mean	3.23	3.18	3.14
Standard Deviation (SD)	.59	.55	.53
Range	1.20–5.10	1.40–5.00	1.53–4.73
Omega^Total^	.84	.87	.90
Omega^Hierarchical (higher order factor, Schmid−Leiman)^	.69	.70	.73
Omega^Specific (variance due to 5 factors)^	.31	.35	.26
Observed Mean inter-item correlation	.154	.158	.153
Observed λ^6^	.797	.878	.901
Projected correlation with full	.76	.84	–
Observed correlation	.87	.97	–
Reliability > .8 across range (IRT θ levels)	– 2.0 to 4.4	– 2.7 to 5.4	– 3.1 to > 6.0
Discrepancy (Short – Long) in points	.09	.04	–
SD of discrepancy	.29	.13	–
95% limits of agreement			
Savings in Length (%)	67%	33%	0%
Standard Error of Measurement	.24	.19	.17
Standard Error of Difference	.34	.28	.24
90% Critical Change	.56	.45	.39
95% Critical Change	.67	.54	.47
Minimal Important Difference (MID, *d* ~ .5)	.30	.27	.26

Observed correlations are based on embedded item administration. Standard errors used ω^Total^ as reliability

*IRT*  Item Response Theory

It also is possible to estimate how accurate the total score is as an overall measure of the items being assessed (Omega^Hierarchical^), as well as how much the total score conveys reliable information about the five underlying specific factors (Withdrawal/Apathy, Thought Disturbance, Aggression, Internalizing, and Delusions/Odd Content) (Omega^Specific^). Table 2 includes these estimates as well. The Omega^Hierarchical^ estimates ranged from 0.69 to 0.73, suggesting that the total score is a mediocre measure of overall severity.

**Fig. 1:**
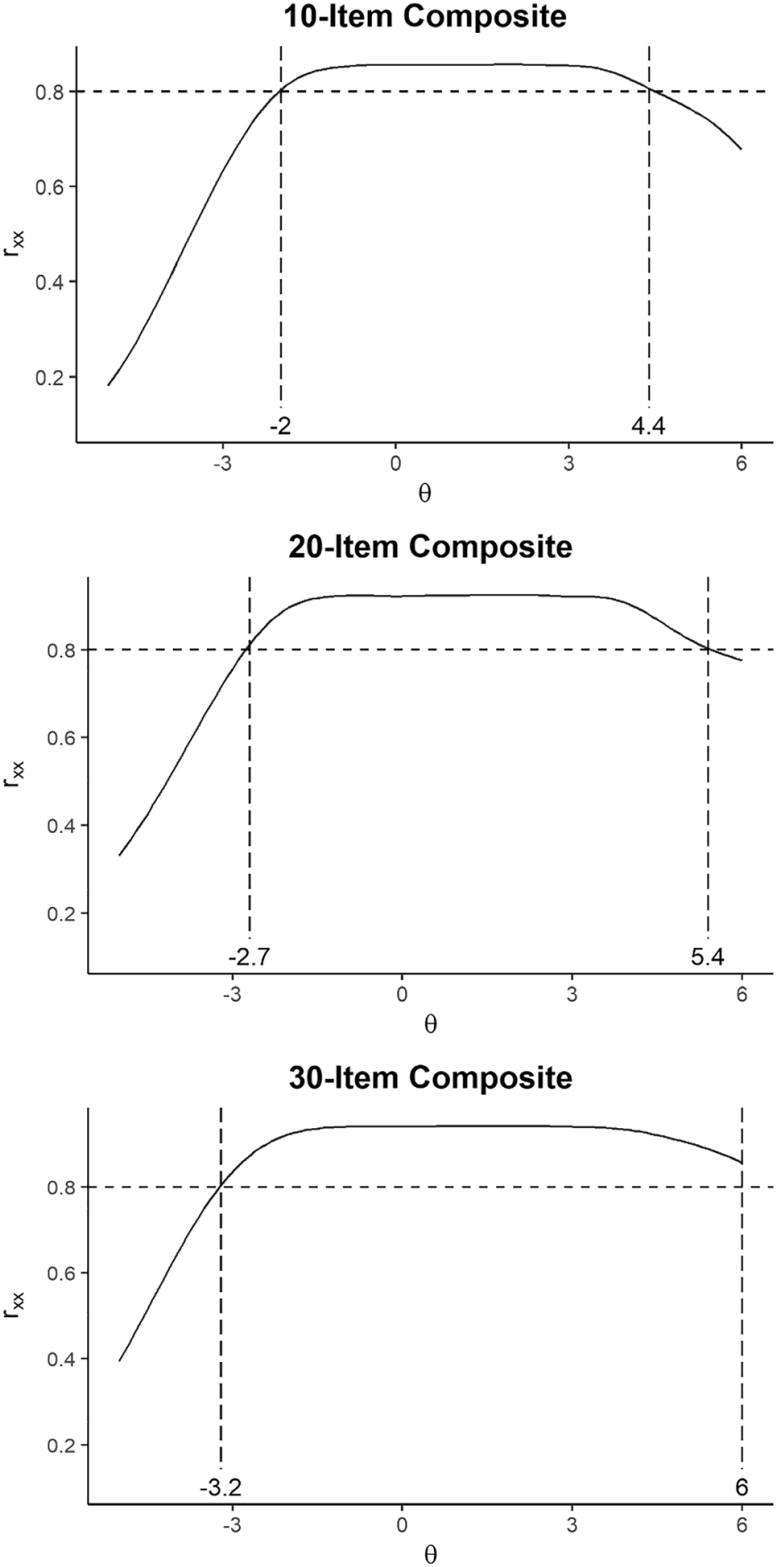
Reliability coverage of composite scores based on graded response model (*N* = 302)

**Fig. 2:**
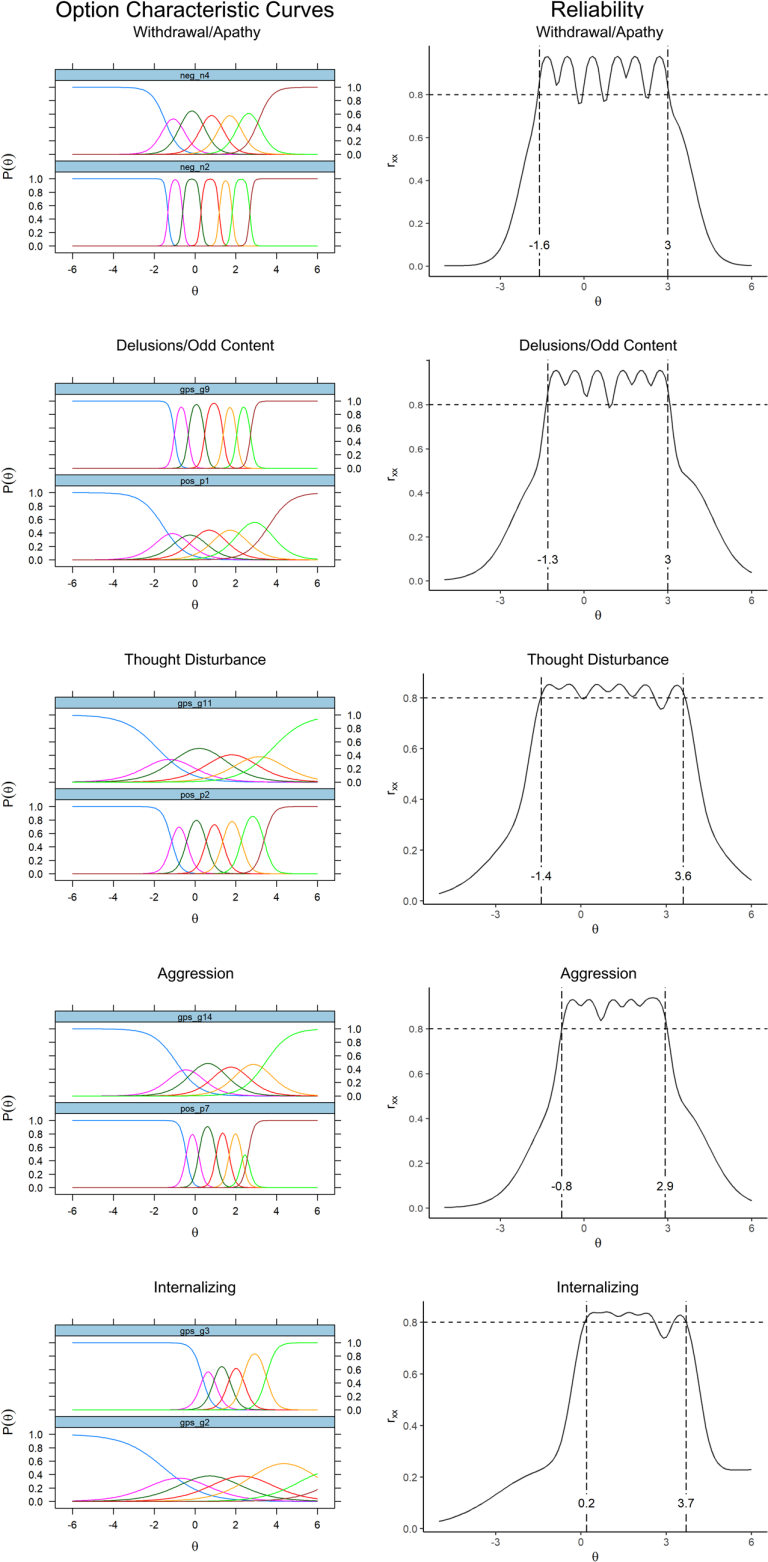
Item Response Theory (IRT) information and reliability estimates for short forms (*N* = 302)*.* From https://en.wikiversity.org/wiki/Evidence_based_assessment/Instruments/PANSS, CC-BY 4.0

Item response theory analyses showed that the PANSS10 composite had reliability > 0.80 between theta levels of – 2.0 to + 4.4 standard deviations above the average trait level (see Fig. 1). The 2-item subscales also showed reliability > 0.80 across a broad severity range (see Fig. 2), even better than found in the original TEOSS sample where we built the 10 and 20 item scales [2]. The PANSS20 form had reliability > 0.80 over an even wider severity range, spanning from – 2.7 to + 5.4. For both the 10- and 20-item analyses, option characteristic curves appeared very good for the subscales. Detailed item option characteristics are available as supplemental tables.

In contrast, IRT analyses of the PANSS30 items found many items with flat information curves (e.g., G1, G3, G6, P4, P5, G10) and implausible parameter estimates. All of these patterns are with the poor loadings in the one-factor CFA model, and also consistent with prior results from both our group [2, 5] and others [14]. Further, reliability for the PANSS30 total was > 0.80 from theta – 3.2 to 6.0. The zone of reliable scores is essentially identical to that offered with the short versions, and in clinical practice, the furthest extremes are unlikely to be encountered. Supplemental materials contain the supporting results and figures.

Table 2 provides the standard errors of measurement (*SEM*) and the difference score (*SE*^*diff*^) for two administrations of the same form, as well as critical values for 90% and 95% confidence differences. These values help determine if a patient's score at two different time points indicates a "reliable change." Furthermore, the table includes a benchmark for the "minimally important difference (MID)," which has been posited to estimate the smallest change that is likely to be considered clinically meaningful [15].

Supplemental analyses checked the reliability coefficients in all available observations, in addition to the baseline scores. The reliability estimates all increased considerably over time, consistent with both theory and prior observations [5] (see supplemental materials). Treatment often increases the variability between patients, both because they have varying treatment response, in addition to enrollment criteria often restricting the range of scores at study entry [16, 17].

### Content coverage, accuracy, and assessment of bias

Content coverage was excellent, *r* = 0.87 for the PANSS10 and 0.97 for the PANSS20 with the full-length scale using the baseline scores, and* r* = 0.94 for the PANSS10 and 0.97 for the PANSS20 with the full-length scale based on all observations across all waves (all *p* < 0.00005). All were larger than the projected correlations estimates based on the internal consistency and reduced scale length, *r*^*hat*^ = 0.76 for a PANSS10 version and 0.84 for the 20 items.

**Fig. 3:**
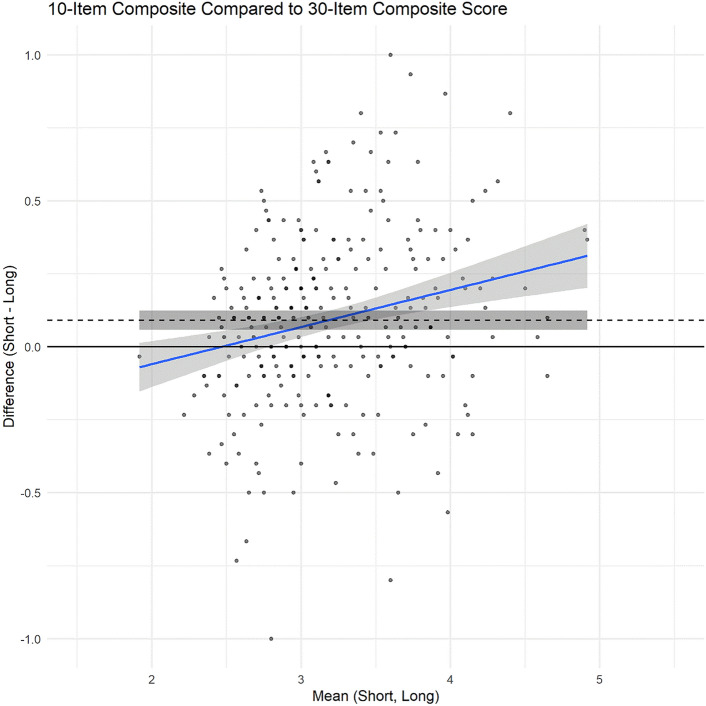
Bland–Altman Plots comparing accuracy of PANSS10 to the PANSS30 scores, *N* = 302. Scores are scaled as item averages, ranging from 1 to 7. Dashed line indicates average bias; blue line is regression

We used regression analyses and Bland–Altman plots to check reproducibility and potential miscalibration or bias comparing the short forms to the full length. Results indicated a slight tendency for short form scores to trend higher than the full length as scores increased; however, the average discrepancy was negligible (i.e., < 0.1 points on average), and only statistically detectable at moderate to high score levels (e.g., item score averages of 3 or higher). Of note, the average discrepancies were smallest in the score range used as an enrollment criterion for the trial (e.g., average discrepancy of zero at observed scores around an item average of 2.0, or a PANSS30 total sum score of 60). Figure 3 shows these results for the PANSS10 (for the PANSS20, see Supplemental Fig. 1). These findings also closely replicate what we found in prior analyses in the earlier referenced paliperidone sample. [5].

### Convergent correlations with CGI-S ratings

Table 3 reports correlations for the three PANSS version totals and the CGI-S, which was the other primary outcome measure in the clinical trial. The PANSS10 and PANSS20 both correlated 0.55 with the CGI-S at baseline, versus the PANSS30 showing a 0.59 correlation. Because of the very high correlations the short forms showed with the full-length PANSS (0.87 and 0.97), the Steiger test of the difference between paired correlations was statistically significant, *t* = 3.60, *p* = 0.0004 for the PANSS20, albeit being too small to be of practical concern. The PANSS10 did not differ significantly in CGI-S correlation versus the 30-item, *t* = 1.61, *p* = 0.109.

**Table 3:** Criterion correlations for full-length, PANSS10 and 20-item pediatric PANSS short forms (*N* = 302, *N* = 1914 observations for change from baseline eta-squared)

	Baseline Criterion Correlations	Eta-squared for LOCF Analyses			
Scale	PANSS30 Total	CGI-Severity	Change from Baseline	Between Treatment Arms	Change x Treatment Arms
PANSS30	1.00	.586****	.227****	.001	.013*
PANSS20	.971****	.545****	.219****	.001	.013*
PANSS10	.872****	.548****	.217****	.005*	.011
Two item scores					
Aggression	.432****	.252****	.093****	.012****	.005
Withdrawal/Apathy	.576****	.335****	.118****	.000	.008
Thought Disturbance	.595****	.367****	.103****	.028****	.005
Internalizing	.336****	.141****	.122****	.019****	.004
Delusions/Odd Content	.511****	.428****	.114****	.005*	.006

**** *p* < .00005, *** *p* < .0005, ** *p* < .005, *p < .05, two-tailed, unless otherwise indicated; LOCF = last observation carried forward

### Sensitivity to change during treatment

The PANSS10, PANSS20, and PANSS30 totals produced essentially identical estimates of treatment effects based on several analyses. Pre-post effect sizes using last observation carried forward (LOCF) yielded eta-squared values for time from 0.22 to 0.23, all *p* < 0.00005, showing large and essentially identical improvement estimates. The main effects for treatment and time-by-treatment were all eta-squared < 0.015 (with all reaching nominal *p* < 0.05 except for time*treatment using the PANSS10, which was *p* = 0.066, before any post hoc correction versions.

## Discussion

The present study serves to replicate and extend initial findings and confirm the utility of our shortened and “optimized” PANSS versions for use in pediatric trials. The shortened PANSS versions we derived based on the NIMH TEOSS pediatric schizophrenia trial dataset [2], which performed as well as the original 30-item PANSS in the TEOSS trial with respect to psychometric integrity and change over time, have now been examined and tested in two additional wholly independent placebo-controlled positive outcome multicenter pivotal trials by different sponsors with different drugs (paliperidone pivotal trial [5] and herein, in this placebo-controlled multicenter aripiprazole pivotal trial. In all instances, the 10-item and 20-item PANSS versions demonstrated psychometric properties that equaled those of the full 30-item version.

Although the benefits of a shorter scale seem obvious with respect to lower burden to adolescent patients, caregivers, and the practitioners who administer the scale, the guiding question of most interest is whether the reduction of items would serve to detract from signal detection. Clearly, pediatric clinical trials that are not optimized for detecting signal are in many ways a waste of precious human resources and a betrayal of the good faith efforts of participants, families, sponsors, regulators, and ultimately the field at large.

For both the paliperidone randomized placebo-controlled trial, and now this aripiprazole randomized placebo controlled trial, we found each shortened version to detect drug vs placebo treatment effects as well as or better than the full 30-item scale.

Results showed good reliability and high correlation between the short forms (10- and 20-item) and the standard 30-item version. No clinical bias was detected, and prorated scores closely matched the full-length form, particularly within the score range commonly used in clinical trials (e.g., total scores of 60–120). Short and full-length form scores also had similar correlations with CGI-S scores.

Results strongly replicated prior work indicating that the PANSS items tap five modestly correlated factors [7–9, 14, 18, 19]. Confirmatory factor analyses found that the 10- and 20- item scales developed in Findling al. [2] retained the consensus five-factor model [7, 14], with all items showing good loadings on the posited factor. Whether using 30, 20, or 10 items, the five-factor model fit markedly better than a one factor model. The total composite score provided high reliability across an extremely wide range of severity levels for all three lengths. The lack of judgment and insight item (G12) showed weak factor loadings and poor item characteristics, also consistent with prior work in adult as well as pediatric samples.

In keeping with the PANSS having multiple underlying factors with low correlations between them, the Guttman lambda^6^ and Omega^Total^ reliability estimates were higher than Cronbach’s alpha. Alpha assumes that all of the items are related to a single underlying factor [10], which is well-established not to be the case for the PANSS. The reliability findings for the five subscales based on both the 10- and 20- item subsets also were good, and even better than we found in prior analyses with the paliperidone dataset [5].

Psychology and medicine have been facing “replication crises” [20, 21]. Practice guidelines, the EQUATOR guidelines, and even the Wikipedia guidelines for articles on medicine-related topics all stress the importance of replication, and de-emphasize findings based on a single study. To address this need for replication before recommending clinical implementation, we have worked to obtain access to multiple large, independent data sets based on registered clinical trials, and we have used consistent statistical methods and an a priori choice of factor structure and items to retain in all our subsequent replications and extensions. In addition, replicating with completely independent samples, the studies used different patients, different countries, different raters, and different pharmacological interventions [22, 23]. Each of these variations heightens the risk of the effect size shrinking. Despite this, the short forms have shown high reliability, convergent validity, and sensitivity to change during treatment that compare favorably to the full-length version.

The current paper has certain limitations, such as being a secondary analysis of a clinical trial where the PANSS was administered in its full 30-item format. To ensure item performance is not affected by contextual factors, it would be beneficial to examine the reliability and validity of the shortened version when administered independently. This would ensure that item characteristics were not dependent on the context created by interviewing about the other (subsequently omitted) items. Because these items performed poorly – not just in present analyses, but also in other pediatric samples, and indeed, across adult samples as well (see Santor et al. [14] for review)—they are unlikely to be contributing crucial context for responses to the stronger, retained items. Using a shortened interview also could reduce the burden and duration, potentially enhancing rater and participant focus and consequently improving scale reliability. Further research is also warranted to explore the reliability and treatment sensitivity of the five subscales.

### Implications

Our work provides further support and confirmation of the utility of the shortened PANSS for pediatric trials. The 10-item and 20-item versions we developed from the TEOSS dataset have performed equivalently to each other and equivalently to the 30-item version in their ability to detect baseline to endpoint treatment change; in addition, we now have 2 large independent placebo-controlled positive drug trials replicating the psychometrics and showing equivalent drug/placebo signal detection for each of the three versions.

Given the rich body of findings, our recommendation for clinical and psychopharmacology trial use at present is that the 10-item version be used. The 10-item version not only reduces burden but performed as well as the 20- and 30-item versions across a wide range of adolescent patients with severity matching that sought in psychopharmacology registration trials. That said, researchers or drug developers targeting symptoms not covered on the 10-item version are always free to use the 20-item or even the full 30-item version if the symptom of interest so requires. We are providing the psychometric analyses and comparisons from all 3 versions (10-item, 20-item, and 30-item) as supplementary data to assist others and in the hopes of further growing the literature on psychometric characteristics of these PANSS versions in pediatric samples.

## Future directions

Efforts to further optimize the PANSS assessment in pediatric trials should continue. To help improve standardization, reduce noise, and improve accuracy, our group is currently developing a pediatric semi-structured interview for the 10-item version to assist clinicians and researchers in assessing the 10 targeted items. Surprisingly, although many of us (JB, DGD, RLF) have long trained investigators in best practices when interviewing adolescents and their parents on the PANSS, a structured interview for the pediatric age group has never been developed. A standardized semi-structured interview should benefit all stakeholders: it would assist clinicians in better assessing symptoms initially and over time, allowing for more informed clinical management, and it would assist researchers by reducing interrater and intrarater variance, thus improving signal detection and allowing for a more robust and reliable determination of treatment effects for the ultimate benefit of our patients and their families.

## Supplementary Information

Below is the link to the electronic supplementary material.

Supplementary file1 (TIF 478 kb)

## Data Availability

This publication is based on research using data from Otsuka that has been made available through Vivli, Inc. Vivli has not contributed to or approved, and is not in any way responsible for, the contents of this publication. The secondary data analyses of the current study are available from the authors. Supplementary data are also available on request.
