# Supplementary figures and images for: Replicating and extending the reliability, criterion validity, and treatment sensitivity of the shortened PANSS for pediatric trials
Source: Eur Child Adolesc Psychiatry. 2025 Mar 10;34(9):2707–16. doi: 10.1007/s00787-025-02681-1 (PMC12507937; doi:10.1007/s00787-025-02681-1)

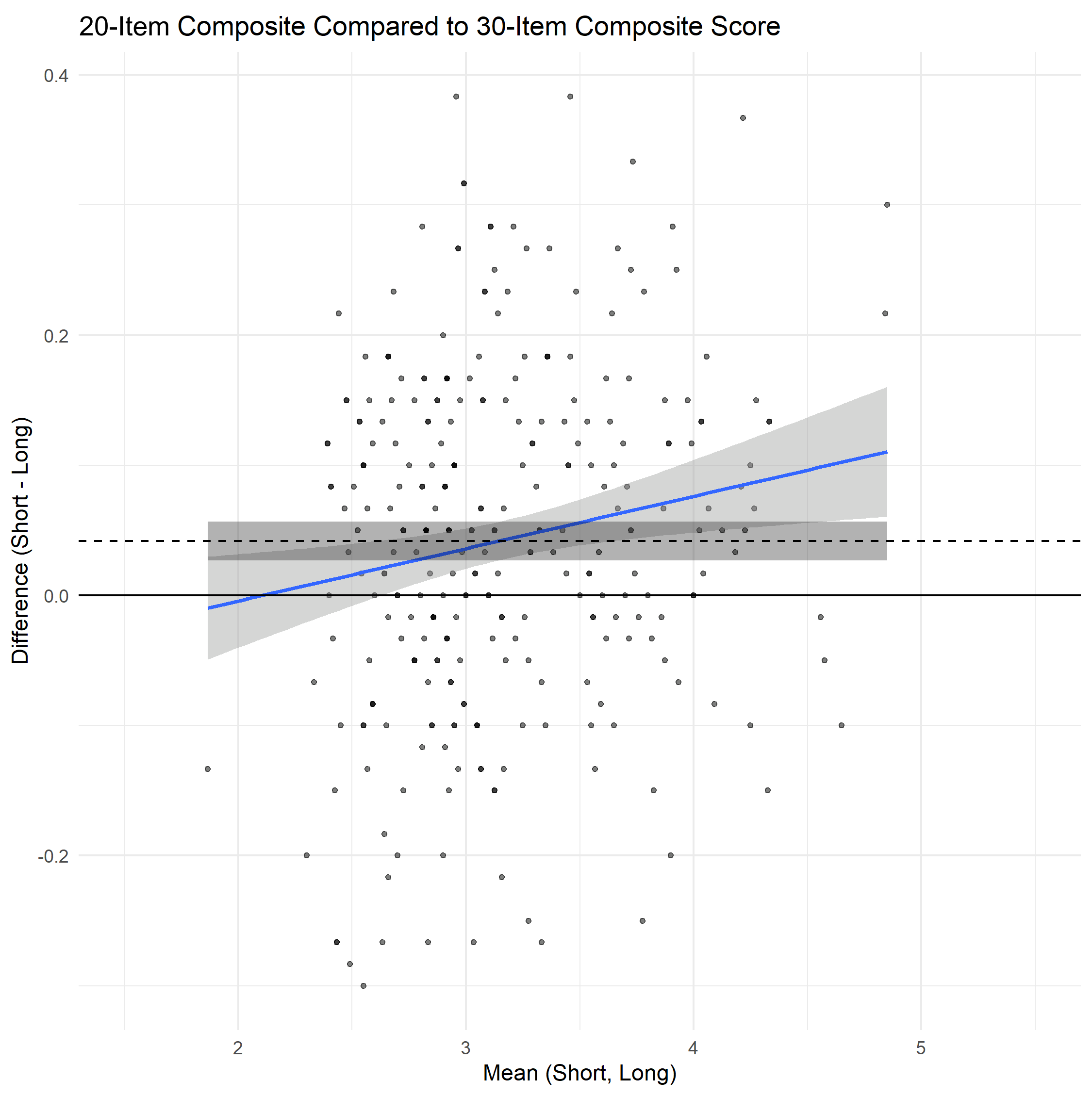

Supplement: Supplementary file 1 — Supplementary file1 (TIF 478 kb) [file 787_2025_2681_MOESM1_ESM.tif]
